# Firmicutes/Bacteroidetes and Firmicutes/Proteobacteria ratios are associated with worse prognosis in a cohort of Latin American patients with cirrhosis

**DOI:** 10.1016/j.clinsp.2024.100471

**Published:** 2024-08-03

**Authors:** Augusto Mantovani, Larisse Longo, Rutiane Ullmann Thoen, Pabulo Henrique Rampelotto, Raul Salinas, Gabriel Tayguara Silveira Guerreiro, Mário Reis Álvares-da-Silva

**Affiliations:** a Graduate Program in Gastroenterology and Hepatology, Universidade Federal do Rio Grande do Sul, Porto Alegre, RS, Brazil; b Experimental Laboratory of Hepatology and Gastroenterology, Center for Experimental Research, Hospital de Clínicas de Porto Alegre (HCPA), Porto Alegre, RS, Brazil; c Bioinformatics and Biostatistics Core Facility, Institute of Basic Health Sciences, Universidade Federal do Rio Grande do Sul, Porto Alegre, RS, Brazil; d Graduate Program in Biological Sciences: Pharmacology and Therapeutics, Universidade Federal do Rio Grande do Sul, Porto Alegre, RS, Brazil; e Conselho Nacional de Desenvolvimento Científico e Tecnológico, CNPq Researcher, Brazil; f Division of Gastroenterology, Hospital de Clínicas de Porto Alegre, Porto Alegre, RS, Brazil

**Keywords:** Alpha-Diversity, Cirrhosis, Gut Microbiota, Gut-Liver axis

## Abstract

**Background:**

Some evidence suggests an association between gut dysbiosis and cirrhosis progression. The authors investigated Gut Microbiome (GM) influence on 90-day mortality and hospitalization/rehospitalization rates in cirrhotic patients.

**Methods:**

Compensated/decompensated outpatients and decompensated inpatients were prospectively included and compared to healthy controls. Clinical, laboratory, GM, and two ratios between phyla were evaluated. Patients were followed up for 90 days for hospitalization/rehospitalization and mortality.

**Results:**

165 individuals were included (50 compensated, 49 decompensated outpatients; 36 decompensated inpatients; 30 healthy), 48.5 % female, mean age was 61, main cirrhosis etiology was hepatitis C (27.3 %), and mostly Child-Pugh (CP) B patients, median MELD of 13. As liver disease progressed, microbiota diversity decreased between the groups (p = 0.05; p < 0.004). There were 9 deaths and 22 hospitalizations or rehospitalizations. GM composition had correlation with norfloxacin (p = 0.36, p = 0.04), encephalopathy (p = 0.31, p = 0.01), lactulose (p = 0.26, p = 0.01), 90-day mortality (p = 0.22, p = 0.04), CP (p = 0.17, p = 0.01), previous 6-month antibiotic use (p = 0.16, p = 0.01), MELD (p = 0.145, p = 0.01), ALBI (p = 0.1, p = 0.04) and 90-day hospitalization/rehospitalization (p = 0.08, p = 0.03). *Firmicutes/Bacteroidetes* (F/B) and *Firmicutes/Proteobacteria* (F/P) ratios were progressively lower and more significant and had an association with 90-day mortality (p < 0.001). Three MELD set-points (≥ 15, 18 and 20) were significantly associated with both ratios, with similar accuracies.

**Conclusions:**

GM dysbiosis was associated with higher CP, MELD, 90-day mortality and hospitalization/rehospitalization. F/B and F/P ratios were associated with 90-day mortality.

## Highlights

• Gut Microbiota (GM) has an important role in liver diseases progression.
• Notable differences in GM and diversity exist between healthy individuals and cirrhosis patients.
• Changes in GM are associated with an increased risk of mortality within 90 days.
• *Firmicutes/Bacteroidetes* and *Firmicutes/Proteobacteria* predict hepatic decompensation and mortality.

## Introduction

As liver fibrosis progresses, there is a reduction in bile flow leading to cholestasis. This impacts the enterohepatic circulation, exerting a notable influence on the Gut Microbiota (GM). Variations in the composition of the gut microbiota can be ascribed to changes in intestinal motility, permeability, barrier function of the lymphatic and blood compartments, portal hypertension, and the immune system.^1^^,^^2^ Consequently, decompensated cirrhosis is not solely secondary to increased intestinal permeability but also to the migration of pathogenic bacteria from the intestine to the lymph nodes and is linked with noteworthy clinical occurrences, including Spontaneous Bacterial Peritonitis (SBP), ascites, Hepatic Encephalopathy (HE), and Acute-on-Chronic Liver Failure (ACLF).^3^ The scrutiny of the mechanisms involved in the development of cirrhosis and the formulation of strategies to hinder and reverse its progression is key, which includes gut-liver axis aftereffects.

Changes in GM can be prompted by medications commonly employed in the management of cirrhosis and other concurrent conditions. In the case of Hepatic Encephalopathy (HE) management, medications such as lactulose and rifaximin, can influence GM composition and its functionality, respectively.[4], [5], [6] The administration of norfloxacin for SBP prophylaxis has also been observed to alter GM composition.^7^ Furthermore, the use of Proton Pump Inhibitors (PPI) has been associated with an increase in oral-located bacteria in GM.^8^ Additionally, the routine use of antibiotics for suspected or confirmed infections in individuals with cirrhosis can also bring about GM modifications.

Recent studies propose a plausible connection between GM dysbiosis to an increased frequency of hospital admissions, and a heightened risk of mortality among individuals with cirrhosis.^7^^,^^9^ Additionally, in patients experiencing decompensated cirrhosis, particularly those with HE, a correlation has been identified with specific families such as *Enterobacteriaceae, Veillonellaceae*, and *Fusobacteriaceae* in GM.^10^ Understanding the gut-liver axis and the factors influencing it is crucial, as it offers potential points of intervention both during the pre-cirrhotic stage and after its onset to prevent decompensations and associated morbidity and mortality.^3^ In line with this, the main objective of the present study was to assess the diversity of the GM composition in a diverse spectrum of Latin American patients diagnosed with cirrhosis, as well as to evaluate 90-day mortality and hospitalization/rehospitalization rates.

## Methods

### Study population and design

A prospective cohort investigation was carried out, encompassing sequential cases of cirrhosis with any causative factor. These cases were drawn from individuals attending both the emergency department and outpatient clinic at Hospital de Clínicas de Porto Alegre, a tertiary-level referral hospital in south Brazil. The study period spanned from March 2018 to February 2019, and participants were categorized into four distinct groups: 1) Compensated outpatients, 2) Decompensated outpatients, 3) Acutely decompensated inpatients, and 4) A control group.

For the inclusion of the control group, the authors enlisted participants of both genders aged 18 years and older. Eligible individuals in this group did not possess a diagnosis of any chronic disease and affirmed abstaining from the use of medications or antibiotics for the six months preceding their inclusion in the study. Healthy individuals were recruited to form the control group with the aim of correlating possible modifications in the gut microbiota composition with compensated cirrhotic patients, reinforcing the need for a marker to differentiate the different stages of cirrhosis progression.

The cirrhotic group inclusion criteria encompassed a confirmed diagnosis validated through clinical, ultrasound, endoscopy, elastography, biopsy, or a composite of these methods; participants had to be over 18 years old. Exclusion criteria comprised hospitalization within 30 days before enrollment, ongoing use of alcohol or illicit drugs (with a minimum abstinence period of six months), presence of hepatocellular carcinoma beyond the Milan criteria, a history of organ transplantation, HIV infection, and pregnancy. The criteria of decompensated cirrhosis for outpatient inclusion were Child-Pugh (CP) scores of 7 or above, a Model for End-stage Liver Disease (MELD) score of ≥ 14 at screening, or clinically evident HE or ascites.^11^ It's worth noting that overt HE (West Haven criteria grade > 1) was considered, as the study did not assess covert HE.

The inpatient group inclusion criteria encompassed admission for more than 24 h due to complications such as ascites, HE, and gastrointestinal bleeding. On the other hand, exclusion criteria included more than 96 h between hospital admission and recruitment and patients admitted for elective procedures. Throughout the hospitalization period, patients were closely monitored to assess progression in line with the European Association for the Study of the Liver (EASL) guidelines for Acute-On-Chronic Liver Failure (ACLF).

This study received approval from the Ethics Committee of Hospital de Clínicas de Porto Alegre (CAAE 25603519900005327 and CAAE 25364119100005327) and adhered to established guidelines for research involving human subjects. The study follows the STROBE Statement and was conducted in accordance with its specific guidelines. Written informed consent was obtained from all patients or their guardians during the screening visit. A follow-up spanning 90 days from the inclusion in the study was conducted through a combination of phone calls, SMS, and chart reviews, due to the heightened risk of hospital readmission observed in these patients.^7^^,^^9^^,^^12^

### Procedures

On the day of the inclusion in the study, demographic, clinical, and laboratory data were promptly recorded for inpatients, and a fecal sample was collected within 96 h of admission. As for outpatients, demographic, clinical, and laboratory information was gathered on the screening day, and the provision of a stool sample took place within the subsequent 10 days.

The collected laboratory and clinical data encompassed a range of parameters, including the etiology of cirrhosis, alcohol consumption and viral serologies. Other variables comprised various biochemical laboratory parameters, and the history of complications (ascites, gastrointestinal bleeding, hepatic encephalopathy, and/or bacterial infections), prior to inclusion and throughout and after hospitalization.

The assessment also involved the calculation of scores such as CP, MELD, MELD-Na, MELD 3.0, and Albumin-Bilirubin (ALBI). Comprehensive details regarding current and past medications, including any specific antibiotic use within the previous 6 months, as well as a record of comorbidities, were also documented.

Fecal sample collection procedures varied for outpatients and inpatients. Outpatients were requested to submit a stool sample within 7 days of the screening visit. All outpatient collections were conducted using sterile flasks and subsequently stored at -80°C until the extraction of DNA and subsequent sequencing.

In the case of inpatients, the collection occurred within the initial 96 h of their arrival at the emergency room, with a median timeframe of 2 days. The same storage and sequencing procedures were applied to the inpatient samples after collection.

For the extraction of DNA and subsequent 16S rRNA sequencing, genomic material was extracted from fecal samples using a commercial kit (QIAmp DNA Stool Mini kit, Qiagen, Hilden, Germany). The hypervariable V4 region of the rRNA gene was amplified through PCR using genomic DNA and the primer pair 515F (5′-GTGCCAGCMGCCGCGGTAA-3′) and 806R (5′-GGACTACHVGGGTWTCTAAT-3′). To combine various samples in the same reaction, the primer-fusion method was employed, with each sample tagged with a distinct barcode attached to the corresponding PCR product. The amplification process utilized Platinum™ PCR SuperMix High Fidelity (Invitrogen, Carlsbad, CA, USA). The resulting products were confirmed through electrophoresis in an agarose gel, purified with the AMPure XP PCR Purification Kit (Beckman Coulter, Brea, CA, USA), quantified using a Qubit™ dsDNA HS Assay Kit (Invitrogen, Carlsbad, CA, USA), and subjected to emulsion PCR using the Ion Chef™ System (Thermo Fisher Scientific, Waltham, MA, USA). Subsequently, the enriched beads were sequenced using the Ion S5™ System (Thermo Fisher Scientific, Waltham, MA, USA) with an Ion 510™ Chip Kit (Thermo Fisher Scientific, Waltham, MA, USA).

For the bioinformatics analysis, the sequence data obtained from the Ion S5™ System underwent processing using a custom pipeline in Mothur v.1.47.0.^13^ Initially, sequences were stripped of barcodes and primers (with no allowance for mismatches), followed by the application of a quality filter to eliminate low-quality reads. Quality control involved trimming low-quality reads, those with incorrect lengths, those containing ambiguous bases, or those with homopolymers longer than 6 base pairs. Identification and removal of potentially chimeric sequences were carried out using VSEARCH.^14^ Additionally, singletons were excluded to mitigate the inclusion of potentially spurious sequences arising from PCR or sequencing errors.

Following these initial quality filtering and trimming steps, the remaining sequences were clustered into Operational Taxonomic Units (OTUs) with a 99 % identity level. Classification against the SILVA v138 reference database at a 97 % similarity threshold was performed, with the removal of sequences labeled as “unknown”, as well as those identified as eukaryotes, mitochondria, and chloroplasts prior to subsequent analysis. OTUs with fewer than ten reads and those present in two or fewer samples were excluded. The resulting OTU table underwent rarefaction to the smallest library size. Further analyses of the sequence dataset were conducted using R v. 4.0.0, leveraging packages such as vegan, phyloseq, ggplot2, and MicrobiomeAnalystR.

For the analysis of microbial communities and statistical evaluation, alpha diversity was assessed using the number of observed taxa, ACE, and the Shannon index. To compare significant differences among bacterial communities (beta diversity), a Principal Coordinates Analysis (PCoA) was conducted. A matrix utilizing the Bray-Curtis dissimilarity metric was calculated for each pair of samples. The ANOSIM multivariate test was employed on the distance matrix to ascertain statistical confidence for the observed sample grouping in PCoA. To identify additional differences among microbial communities, clustering methods based on Bray-Curtis dissimilarity were executed. Hierarchical clustering results were visualized using dendrograms, and a Venn dendrogram was generated using InteractiVenn.^15^ Differentially abundant taxa at the phylum, family, and genus levels were identified using the Linear Discriminant Effect Size (LEfSe) method.^16^ This method employs a nonparametric factorial Kruskal-Wallis sum rank test and Linear Discriminant Analysis (LDA) to determine statistically significant features among taxa and estimate the effect size of the difference. Differences were considered significant for a logarithmic LDA score threshold of ±1.5 and p < 0.05. After data completion, the *Firmicutes/Bacteroidetes* Ratio (FBR) and *Firmicutes/Proteobacteria* Ratio (FPR) were calculated as objective dysbiosis measures.

Statistical analyses included the Student's *t*-test for normally distributed continuous variables and the Kruskal-Wallis test for non-normally distributed continuous variables. Fisher's Exact test was employed for categorical variables. Spearman's rank correlation coefficient test (ρ) was used to assess correlations between variables. The evaluation of mortality and hospitalization/rehospitalization outcomes associated with phyla ratios was conducted through Receiver Operating Characteristic (ROC) curve analysis. A significance level of 0.05 (alpha error, p-value) was considered for all analyses.

## Results

### Characteristics of the study population

The demographic, clinical, and laboratory data of the enrolled patients are summarized in Table 1. The control group, comprising 30 individuals, had a mean age of 51.6, with 27 % being female, and none presented comorbidities or were using medications. Globally, 84 % were classified as having white skin, while 16 % were of African descent. The most prevalent cause of cirrhosis was hepatitis C infection, accounting for 27.3 % of cases, with 84 % of these patients exhibiting sustained virologic response at enrollment. The median CP score was 8 (interquartile range 6‒13). Proton Pump Inhibitor (PPI) use significantly decreased in accordance with cirrhosis severity. All patients were successfully followed up for 90 days or until death, with no losses in follow-up.

**Table 1:** Enrolled patients' demographic, clinical and laboratory data (Total n = 165).

Variables	Compensated Outpatients (n = 50)	Decompensated Outpatients (n = 49)	Decompensated Inpatients (n = 36)
Age (years old)	61 (24–86)	62 (22–83)	59 (31–80)
Female	25 (50 %)	18 (37 %)	15 (42 %)
Diabetes	19 (38 %)	20 (41 %)	15 (42 %)
BMI (kg/m²)	28.6 (19.3–50.6)	29.6 (18.5–36.3)	26.2 (20.7–39.5)
Cirrhosis Etiology			
HCV	19 (38 %)	13 (26 %)	13 (36 %)
Alcohol	4 (8 %)	12 (24 %)	10 (27 %)
NASH	8 (16 %)	7 (14 %)	3 (8 %)
HCV + alcohol	9 (18 %)	5 (10 %)	4 (11 %)
Other	10 (20 %)	12 (24 %)	6 (17 %)
Hepatocellular Carcinoma	0	0	4 (11 %)
Ascites	0	34 (69 %)	25 (69 %)
Overt Encephalopathy	0	9 (18 %)	19 (53 %)
Creatinine (mg/dL)	0.84 (0.55–1.41)	0.82 (0.56–8.32)	1.0 (0.5–6.0)
Total Bilirubin (mg/dL)	0.8 (0.3–2.6)	2 (0.3–10.1)	1.75 (0.3–13.6)
INR	1.1 (0.9–1.8)	1.4 (0.9–2.9)	1.4 (0.9–3.3)
Albumin (mg/dL)	4.25 (3.4–5.1)	3.2 (2.1–4.3)	3 (2.1–4.3)
Sodium (mg/dL)	140 (134–149)	140 (130–146)	137 (131–150)
Platelets (×10^9^/L)	99 (32–280)	93 (12–212)	88 (22–353)
Child-Pugh	5 (5–6)	8 (7–13)	9 (7–13)
MELD	9 (6–15)	15 (6–25)	15 (7–34)
MELD-Na	9 (6–15)	15 (6–25)	16 (6–34)
MELD 3.0	9 (7–16)	16 (8–26)	17 (7–42)
ALBI	-3.02 (-4.18–-1.98)	-1.83 (-2.91–-0.48)	-1.67 (-3.16–-0.34)
Beta-blockers	23 (46 %)	42 (85 %)	20 (55 %)
Norfloxacin	0	0	4 (11 %)
Rifaximin	1 (2 %)	5 (10 %)	1 (2 %)
Lactulose	1 (2 %)	14 (28 %)	11 (30 %)
PPI	17 (34 %)	14 (28 %)	7 (19 %)
Previous 6-month antibiotic use	11 (22 %)	15 (31 %)	20 (55 %)
Antibiotic treatment at fecal sample collection^a^	1 (2 %)	5 (10 %)	22 (61 %)

Values are median (interquartile range) for quantitative variables and n (%) for qualitative variables. ALBI, Albumin to Bilirrubin Grade; BMI, Body Mass Index; HCV, Hepatitis C Virus; NASH, Non-Alcoholic Steatohepatitis; INR, International Normalized Ratio; MELD, Model for End-Stage Liver Disease; PPI, Proton Pump Inhibitors.

a In hospitalized patients, it refers to treatment received between hospital admission and fecal sample collection for microbiome analysis (14 used a cephalosporin, 3 used a carbapenem) or patients using prophylactic treatment before admission (4 using norfloxacin and 1 using rifaximin).

Among the participants, only two individuals, both belonging to the inpatient group, met the criteria outlined in the EASL guidelines for ACLF. The study recorded a total of nine deaths, all of which were liver-related ‒ seven occurred among inpatients, and two were observed in decompensated outpatients. Additionally, there were 22 instances of hospitalization or rehospitalization, with 11 cases attributed to ascites, 8 to HE, and 3 to gastrointestinal bleeding.

### Gut microbiome composition

In the stool analysis, identification revealed the presence of 12 bacterial phyla, 77 families, and 212 genera. The phyla with the highest relative abundance were *Firmicutes, Bacteroidetes, Proteobacteria, Actinobacteriota*, and *Verrucomicrobia*, listed in descending order of prevalence (Supplementary Figs. 1‒3).

### Composition of the microbiota within and between groups

**Fig. 1:**
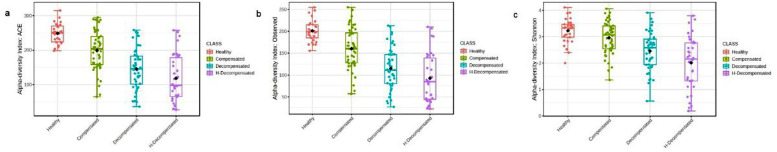
(a–c) Alpha diversity index measured by ACE, observed and Shannon (p < 0.001 for all).

There was a gradual decrease in alpha diversity, as measured by the Shannon and ACE indices, corresponding to the progression in the severity of liver disease. In other words, more severe liver disease was associated with lower bacterial diversity within it (Fig. 1). The statistical analysis indicated a significant difference with p < 0.001 for all measurements. Additionally, substantial differences were noted in beta diversity, signifying considerable dissimilarity in the composition of the microbiota among the various groups (PCoA Bray-Curtis: *R* = 0.10802; p < 0.001).

### Gut microbiome in cirrhotic patients versus the control group

The analysis of predominant phyla revealed that in both the control group and compensated patients, *Firmicutes* was the predominant phylum followed by *Bacteroidetes*. The key families in these groups were *Ruminococcaceae* (LDA = 2.9), *Lachnospiraceae* (LDA = 2.6), and *Prevotellaceae* (LDA = 2.5). As cirrhosis progressed, there was an increase in the *Proteobacteria* phylum, particularly from the *Enterobacteriaceae* family (LDA = 2.6). Conversely, in the decompensated groups, despite *Bacteroidetes* being the main phylum, primarily due to a reduction in *Firmicutes,* there was a progressive decrease in *Oscillospiraceae* (LDA = 2.1) and *Clostridia* (LDA = 1.7).

Regarding bacterial genera, the progression of liver disease was associated with a significant reduction in four representatives of the phylum *Firmicutes*, including *Faecalibacterium* (LDA = 2.9), *Lachnospiraceae* (LDA = 2.1), *Ruminococcaceae* (LDA = 2.0), and *Oscillospirales* (LDA = 2.0).

### Gut microbiome within cirrhotic patients

In the comparison between decompensated and compensated groups, distinct microbial changes were observed. There was a reduction in *Firmicutes* and a notable increase in *Bacteroidetes, Proteobacteria* (primarily from the *Enterobacteriaceae* family ‒ LDA = 2.8), *Fusobacteriota* (especially the *Fusobacteriaceae* family, LDA = 1.5), and *Actinobacteria*, particularly in inpatients. Additionally, there was an increase in the *Veillonellaceae* family (LDA = 1.9), with higher levels in decompensated outpatients.

The study revealed significant associations, measured by Spearman's correlation (ρ), between several clinical and laboratory measurements, shown in Table 2. No significant influence on the microbiota composition was observed for the use of PPI (ρ = -0.03, p = 0.83) or rifaximin (ρ = 0.07, p = 0.16). Importantly, death within 90 days (ρ = 0.22, p = 0.04) and hospitalization/rehospitalization within 90 days (ρ = 0.08, p = 0.03), were directly associated with altered GM.

**Table 2:** Correlation between gut microbiota and medications, clinical scores, and outcomes.

Variables	Spearman's correlation (ρ)	p-value
Norfloxacin	0.36	0.04
Lactulose	0.26	0.01
Antibiotics in the previous 6-months	0.16	0.01
Encephalopathy (present or < 12-months)	0.31	0.01
Child-Pugh score	0.17	0.01
MELD score	0.145	0.01
Proton pump inhibitor	-0.03	0.83
Rifaximin	0.07	0.16
Death within 90-days	0.22	0.04
Hospitalization/rehospitalization within 90-days	0.08	0.03

Correlation between variables was assessed with Spearman's rank correlation coefficient test (ρ). A significance level of p < 0.05 was considered for all analyses. MELD, Model for End-Stage Liver Disease.

**Fig. 2:**
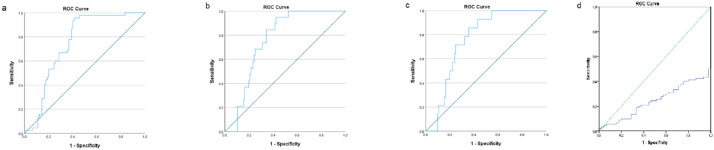
(a‒d) Area Under the Curve (AUC) for three MELD set-points (≥ 15, ≥ 18, ≥ 20 and the control curve of MELD < 15) and *Firmicutes/Bacteroidetes* ratio, respectively 0.747, 0.758 and 0.760, all with p < 0.01 and no difference between set-points (p > 0.05). For MELD < 15, no area under the curve for consideration.

**Fig. 3:**
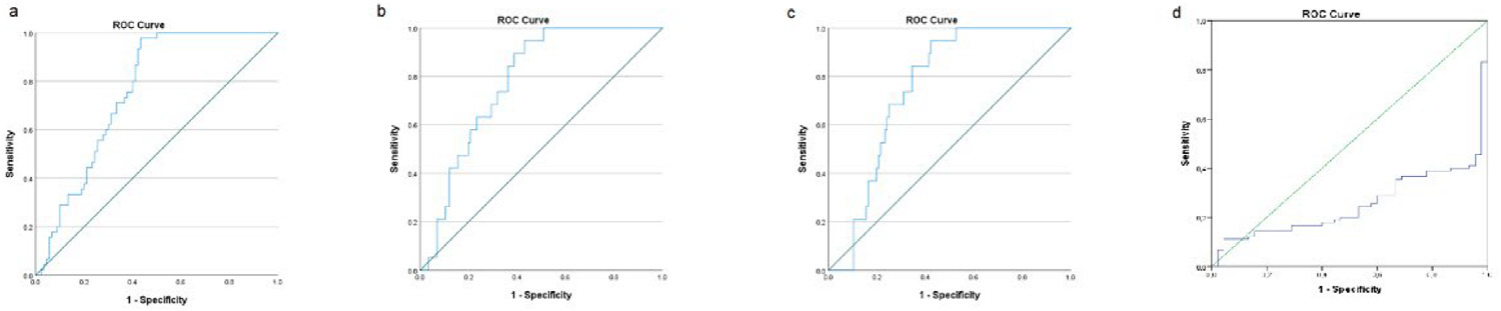
(a‒d) Area Under the Curve (AUC) for three MELD set-points (≥ 15, ≥ 18, ≥ 20 and the control curve of MELD < 15) and *Firmicutes/Proteobacteria* ratio, respectively 0.756, 0.781 and 0.760, all with p < 0.01 and no difference between set-points (p > 0.05). For MELD < 15, no area under the curve for consideration.

**Fig. 4:**
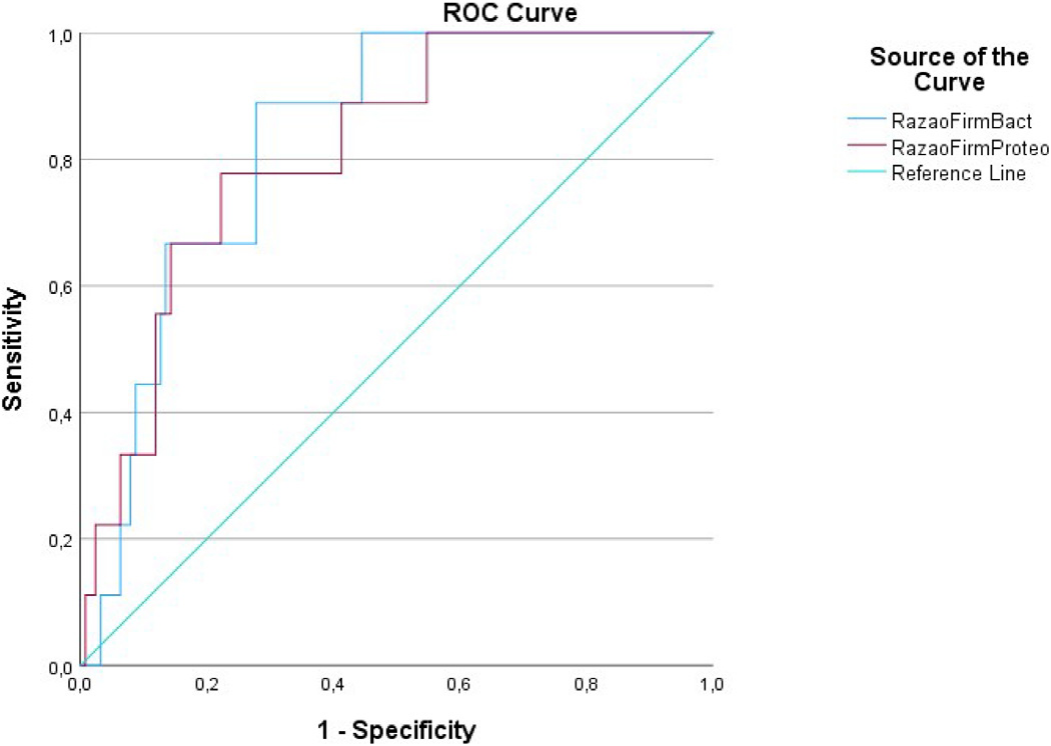
90-day mortality ROC Curve; area under the curve of 83 % (p = 0.01) and 82 % (p = 0.02) for the *Firmicutes/Bacteroidetes* and *Firmicutes/Proteobacteria* ratios, respectively.

The study presented phyla ratios, FBR, and FPR, as shown in Table 3. These ratios objectively indicate GM dysbiosis and the risk of decompensation between the groups, with p < 0.001 for all comparisons. Also, the authors compared these ratios with three set points in MELD scores (≥ 15, ≥ 18 and ≥ 20) to compare accuracy between area under the curve in ROC curves (Fig. 2, Fig. 3, respectively), all with significant p-values, but with no difference between set points. Furthermore, low values of both ratios were associated with 90-day mortality, with an area under the curve of 0.83 (p = 0.01) for FBR and 0.82 (p = 0.02) for FPR, as depicted in Fig. 4.

**Table 3:** Firmicutes/Bacteroidetes and firmicutes/Proteobacteria ratios.

Variables	Control	Compensated outpatients	Decompensated outpatients	Decompensated inpatients
*Firmicutes/Bacteroidetes* ratio	2.6499	1.6464	0.100479	0.09246755
*Firmicutes/Proteobacteria* ratio	72.7557	13.5483	0.847212	0.219197123

Comparison between groups evaluated with the Kruskal-Wallis test with adjustment through Bonferroni correlation. * p < 0.001 for all comparisons between groups.

## Discussion

Cirrhosis is associated with local and systemic immunological, vascular, and inflammatory alterations, which are linked to intestinal barrier dysfunction and intestinal dysbiosis. Disturbances in the gut-liver axis are directly involved in the pathogenesis of compensated cirrhosis and in the type and severity of complications in decompensated cirrhosis, such as bacterial infections, encephalopathy, extrahepatic organ failure, and progression to acute or chronic liver failure. Based on this, this study was the first to evaluate the relationship between intestinal microbiota diversity and the evolution of cirrhosis in a Latin American cohort.

The authors demonstrated notable differences in the GM between healthy individuals and patients with compensated, decompensated cirrhosis, and those hospitalized for hepatic decompensation. Regarding microbial diversity, there was a progressive reduction in *Firmicutes* and an increase in *Bacteroidetes* and *Proteobacteria*. This pattern led to a practical division into two groups: controls and compensated outpatients exhibited a GM predominance of *Firmicutes*, while decompensated patients (outpatients and hospitalized) had a GM predominance of *Bacteroidetes*, with a progressive increase in *Proteobacteria* across the four groups. This study reinforces the association between dysbiosis and the risk of hepatic decompensation. The prominence of the *Firmicutes* and *Bacteroidetes* phyla, with their proportions serving as a marker of microbial dysbiosis, underscores the importance of GM composition in liver disease.^17^ The *Firmicutes* phylum's role in synthesizing short-chain fatty acids, known for stabilizing the intestinal barrier, adds a mechanistic understanding to the observed dysbiosis.^17^ The traditional ratio involving *Firmicutes* and *Bacteroidetes*, studied in various comorbidities and confirmed in this study for all comparisons between groups, provides a valuable tool for assessing GM health.[18], [19], [20] Considering that the *Firmicutes*-predominant GM cluster unifies two very different clinical scenarios, although without cirrhosis complications, suggests the need for an objective differentiation method. This relationship, particularly represented by the FBR, emerges as a practical tool for predicting the risk of hepatic decompensation. The observed decrease in FBR with the progression of liver disease and its association with mortality at 90 days underscore the potential clinical significance of this ratio as a prognostic indicator in individuals with liver disease.

The present study reasserts the association between dysbiosis and the hepatic decompensation risk. The two major phyla were *Firmicutes* and *Bacteroidetes*, and the analysis of the proportion of these two dominant phyla can be used as a marker of microbial dysbiosis.^17^ The *Firmicutes* phylum is primarily responsible for the synthesis of short-chain fatty acids, with one of its properties being the stabilization of the intestinal barrier.^17^ There is a traditional ratio involving *Firmicutes* and *Bacteroidetes*, already studied in the literature in several comorbidities,[18], [19], [20] which was also relevant in this study for all comparisons between groups. Considering that the *Firmicutes*-predominant GM cluster unifies two very different clinical scenarios (healthy individuals and patients with compensated cirrhosis), although no cirrhosis complications were involved, an objective way to clarify this difference is required. This relationship can be considered a practical tool to predict the risk of hepatic decompensation. As demonstrated in this study, with the progression of liver disease, the authors observed a decrease in FBR, with an association of this alteration with mortality at 90 days.

Observing a continuous rise in *Proteobacteria* and considering previous studies that identified an association between more severe hepatopathy and an increased proportion of *Proteobacteria*,^21^^,^^22^ the authors introduced the novel FPR. This ratio exhibited a consistent decrease across all groups, notably diminishing in outpatients transitioning from compensated to decompensated states. Additionally, there was a fourfold reduction between decompensated outpatients and inpatients. Like FBR, FPR demonstrated an association with 90-day mortality. Although FBR and FPR exhibited comparable ROC curve performances (Fig. 4), the absolute decrease proportion in FPR is greater than that in FBR. Consequently, FPR holds the potential to be a more precise ratio for predicting the risk of hepatic decompensation and mortality, warranting further exploration in subsequent studies.

In this study, a substantial decrease in alpha diversity, providing insights into the diversity of microbiota composition within a specific sample, and a significant reduction in beta diversity were demonstrated. These findings align with other studies that have reported an overall decline in *Firmicutes* and an increase in *Bacteroidetes*.^7^^,^^17^ Notably, certain genera associated with disease severity, such as *Clostridiales, Faecalibacterium*, and *Lachnospiraceae*, exhibited the highest LDA in the present study, suggesting a pattern that may be generalized. Previous research has consistently indicated that the progression of liver disease correlates with reduced bacterial diversity rates.^7^^,^^23^ Furthermore, a study conducted by the present research group revealed that Brazilian cirrhotic patients adhering to a diet richer in cereals and yogurt, which was associated with higher microbial diversity, exhibited a lower risk of hospitalizations within a 90-day follow-up period compared to an American cohort consuming a Western diet.^9^

A noteworthy association was observed between HE and the use of lactulose, a finding consistent with prior studies.^4^^,^^9^^,^^24^ However, in this study, the specific contribution of each of these variables remains unclear. A substantial majority (82 %) of patients using lactulose also had a history of past or current HE, with 50 % presenting with current HE at the screening. Notably, only two patients with a history of HE were not using lactulose. This interdependence between lactulose and the presence of HE underscores the challenge of disentangling the individual impact of these factors on the altered GM in the studied cohort.

Among the variables examined, prophylactic norfloxacin exerted the most significant impact on GM composition. In contrast, rifaximin did not exhibit notable differences in GM, aligning with other studies that have linked clinical benefits to the reduction of hyperammonemia and endotoxemia without substantial changes in microbial composition.^5^^,^^6^ Additionally, antibiotic use within the six months preceding the study, including cases where antibiotics were taken shortly before stool collection, was associated with modifications in GM. As expected, the frequency of antibiotic use increased progressively with the severity of the disease. As demonstrated in another comprehensive study,^7^ the use of PPIs did not exhibit any significant association with GM modification. In the present study, a notable reduction in the frequency of PPI use was observed with the progression of cirrhosis, a practice deemed clinically beneficial. This reduction in PPI use may provide an explanation for the lack of association between PPIs and GM modifications in the studied cohort.

The present study has several limitations, including a relatively low number of deaths (6.6 % considering all cirrhotic) and an insufficient number of patients progressing to ACLF to form a specific group. A considerable proportion of patients had used antibiotics in the previous six months, which can alter GM composition, although this was observed in a similar proportion among outpatients. Inpatients received antibiotics more frequently due to proven or suspected bacterial infections, a common aspect in the management of decompensated cirrhosis. Like many microbiome studies, ours was conducted at a single center, raising uncertainties about the generalizability of the present findings.

This was the first study conducted in a Latin American cohort of patients diagnosed with cirrhosis, and the data obtained suggest a direct correlation between the degree of dysbiosis and disease severity. The progressive modification in microbiota composition, reflected in the proportions among phyla, is notably associated with an increased risk of 90-day mortality. FBR and FPR ratios may be used as tools to predict the risk of hepatic decompensation and mortality, ensuring further exploration in subsequent studies. Additionally, these observations indicate that clinical scores, the presence of HE, and the use of specific medications are linked to changes in microbiota composition, suggesting a mutual influence between cause and effect. The present data reveal significant alterations in GM composition related to cirrhosis and its progression with clinical outcomes such as mortality and hospitalization within 90 days. Considering the pivotal role of specific GM alterations in liver disease progression, exploring therapeutic possibilities for microbiota modulation has become a significant focus in modern hepatology. In the future, larger, preferably multicenter studies should be conducted to enhance the data and reliability of the findings presented in the current study.

## Authors’ contributions

Mantovani A and Álvares-da-Silva MR performed the conceptualization, methodology, formal analysis, investigation, data curation, writing of the original draft, writing-review, and editing; Longo L, Thoen RU, Salinas R, and Guerreiro GTS performed the methodology, investigation, writing review and editing; Rampelotto PH performed the bioinformatics analysis.

## Declaration of competing interest

The authors declare no conflicts of interest.

## Appendix Supplementary materials

Image, application 1
