# Supplementary material for: Firmicutes/Bacteroidetes and Firmicutes/Proteobacteria ratios are associated with worse prognosis in a cohort of Latin American patients with cirrhosis
Source: Clinics (Sao Paulo). 2024 Aug 3;79:100471. doi: 10.1016/j.clinsp.2024.100471 (PMC11345307; doi:10.1016/j.clinsp.2024.100471)

**CLINICS-D-24-00303_Supplementary Material**

**Supplementary Figure 1** (a–d) Phyla composition in controls, compensated and decompensated outpatients, and decompensated inpatients.


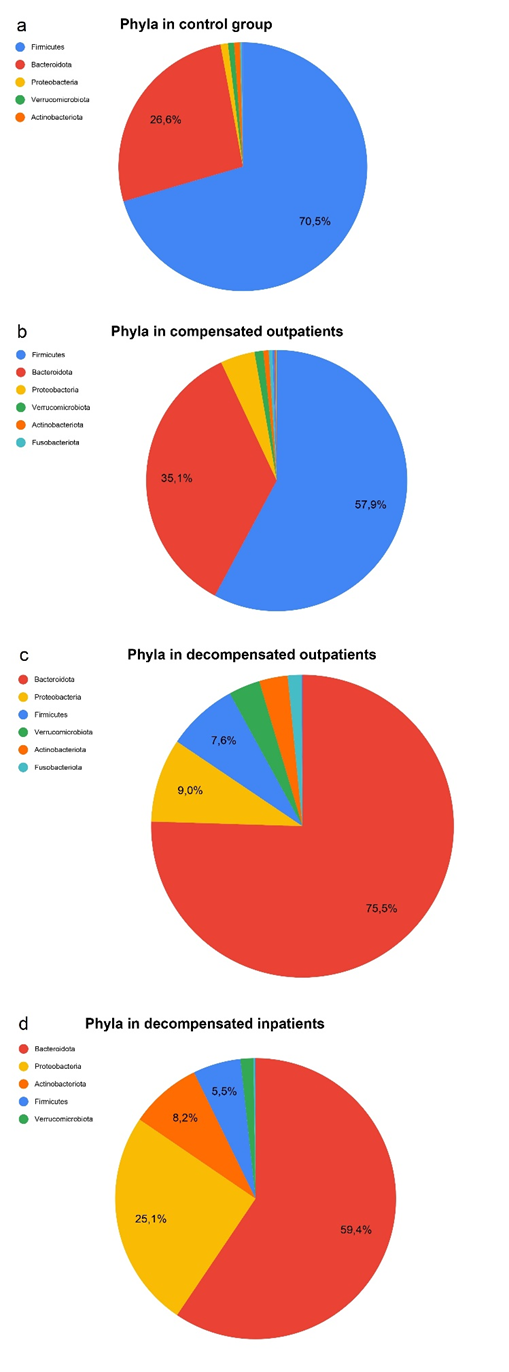


**Supplementary Figure 2** (a–d) Family composition in controls, compensated and decompensated outpatients and decompensated inpatients.


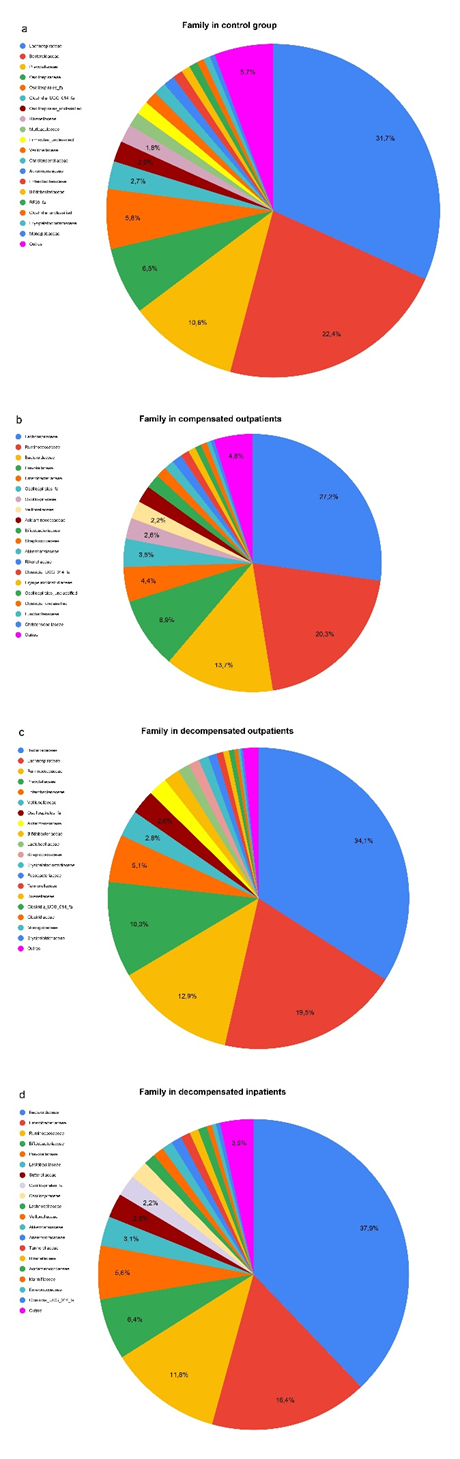


**Supplementary Figure 3** (a–d). Genera composition in controls, compensated and decompensated outpatients and decompensated inpatients.


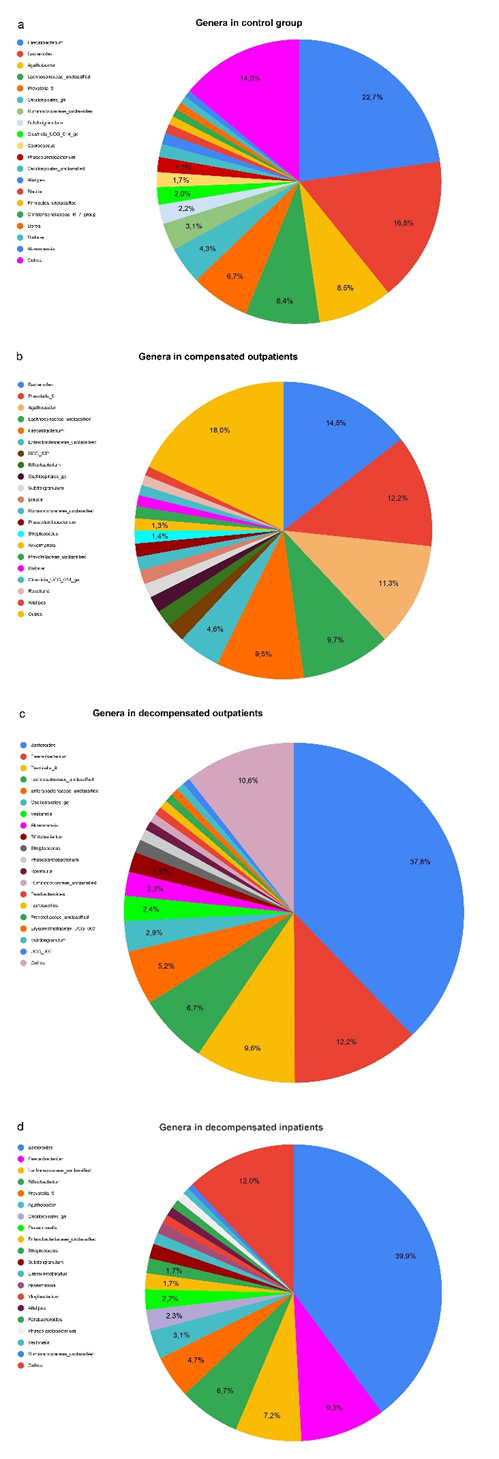

Supplement: Supplementary file 1 [file mmc1.docx]
